# PDE10A Inhibitors—Clinical Failure or Window Into Antipsychotic Drug Action?

**DOI:** 10.3389/fnins.2020.600178

**Published:** 2021-01-20

**Authors:** Frank S. Menniti, Thomas A. Chappie, Christopher J. Schmidt

**Affiliations:** ^1^George & Anne Ryan Institute for Neuroscience, University of Rhode Island, Kingston, RI, United States; ^2^Internal Medicine Medicinal Chemistry, Pfizer Worldwide Research and Development, Cambridge, MA, United States; ^3^Pfizer Innovation and Research Lab Unit, Pfizer Worldwide Research and Development, Cambridge, MA, United States

**Keywords:** schizophrenia, PDE10A, basal ganglia, dopamine, cyclic nucleotide phosphodiesterase, medium spiny neuron, antipsychotic action

## Abstract

PDE10A, a phosphodiesterase that inactivates both cAMP and cGMP, is a unique signaling molecule in being highly and nearly exclusively expressed in striatal medium spiny neurons. These neurons dynamically integrate cortical information with dopamine-signaled value to mediate action selection among available behavioral options. Medium spiny neurons are components of either the direct or indirect striatal output pathways. Selective activation of indirect pathway medium spiny neurons by dopamine D2 receptor antagonists is putatively a key element in the mechanism of their antipsychotic efficacy. While PDE10A is expressed in all medium spiny neurons, studies in rodents indicated that PDE10A inhibition has behavioral effects in several key assays that phenocopy dopamine D2 receptor inhibition. This finding gave rise to the hypothesis that PDE10A inhibition also preferentially activates indirect pathway medium spiny neurons, a hypothesis that is consistent with electrophysiological, neurochemical, and molecular effects of PDE10A inhibitors. These data underwrote industry-wide efforts to investigate and develop PDE10A inhibitors as novel antipsychotics. Disappointingly, PDE10A inhibitors from 3 companies failed to evidence antipsychotic activity in patients with schizophrenia to the same extent as standard-of-care D2 antagonists. Given the notable similarities between PDE10A inhibitors and D2 antagonists, gaining an understanding of why only the latter class is antipsychotic affords a unique window into the basis for this therapeutic efficacy. With this in mind, we review the data on PDE10A inhibition as a step toward back-translating the limited antipsychotic efficacy of PDE10A inhibitors, hopefully to inform new efforts to develop better therapeutics to treat psychosis and schizophrenia.

## Introduction

**Figure 1:**
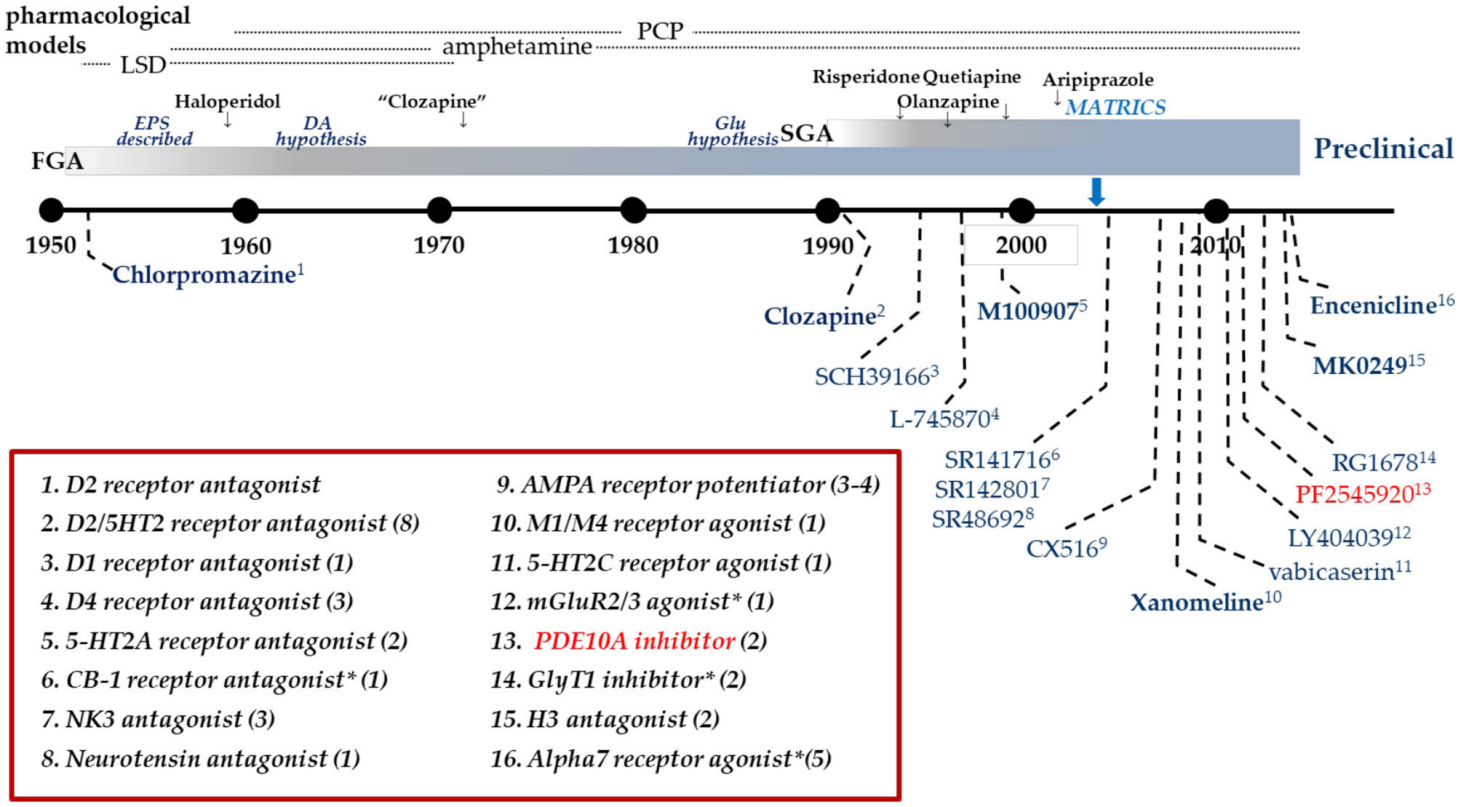
Clinical drug development in Schizophrenia. Timeline of clinical drug development in schizophrenia following the discovery of the antipsychotic activity of chlorpromazine in 1952. Individual compounds are listed in the graphic with their pharmacological mechanism identified in the box. Many mechanisms have been targeted with multiple compounds as identified by the value in parentheses. Dates are an approximation, based on publication dates or drug approval. *Includes allosteric modulators.

Dopamine D2 receptor antagonists have been the standard of care pharmacotherapy for the treatment of psychosis in schizophrenia since the 1950's. In the intervening decades, there has been considerable research seeking to gain insight into the molecular basis for the antipsychotic mechanism of these drugs. A significant contribution to this effort has been the development of pharmaceutical agents directed at alternative molecular targets and their clinical testing for antipsychotic efficacy (Figure 1). However, of the 14 mechanisms listed in Figure 1, only one, the muscarinic M1-selective agonist xanomeline (Shekhar et al., 2008), approached the efficacy of D2 antagonists. Certainly none of the tested mechanisms evidenced superiority to the standard of care. What has largely been missing from this effort is the back-translation of the molecular pharmacology of the tested-but-failed agents or classes of agents. Simply put, how did these agents affect the brain similarly and yet differently than D2 receptor antagonists to give insight into the nature of antipsychotic drug action? Recently, there has been an industry-wide effort to develop and test inhibitors of phosphodiesterase 10A (PDE10A) as a novel mechanism to ameliorate psychosis (Chappie et al., 2012; Jørgensen et al., 2013; Geerts et al., 2017; Jankowska et al., 2019). Several PDE10A inhibitors were tested in various settings in patients with schizophrenia. While some signs of efficacy was noted on measures of global clinical impressions in one study (Macek et al., 2019), overall these compounds failed to demonstrate convincing evidence of benefit equivalent to the standard of care D2 antagonists (DeMartinis et al., 2019; Macek et al., 2019; Walling et al., 2019). Moving forward from these disappointing results, comparing and contrasting the effects of PDE10A inhibitors with D2 antagonists provides a new opportunity for back translational research to gain insight into factors critical to the molecular basis of antipsychotic drug action. The fact that PDE10A inhibitors have an unusually precise molecular pharmacology, the enzyme is restricted to striatal medium spiny neurons and inhibitors increase cyclic nucleotide levels only in these neurons, may be particularly advantageous to such efforts. With this in mind, we review the data on PDE10A inhibition as a step toward such back-translation, hopefully to inform new efforts to develop better therapeutics to treat psychosis and schizophrenia.

Phosphodiesterase 10A (PDE10A) belongs to the phosphodiesterase superfamily of enzymes that control cAMP and cGMP signaling within cells throughout the body (Conti and Beavo, 2007). This control of cyclic nucleotide signaling is accomplished through the hydrolysis of signaling-capable cAMP or cGMP to signaling-silent AMP or GMP. PDEs are differentially and dynamically localized at the cellular and subcellular levels to control the intensity, direction, and longevity of cyclic nucleotide signaling engaged by external stimulation of G-protein coupled receptors and Ca^2+^ signaling mechanisms. Given the key regulatory role of PDEs in cellular communication, pharmacological manipulation has proven to be an attractive avenue for development of drugs to treat various human diseases (Lugnier, 2006; Menniti et al., 2006; Baillie et al., 2019). Inhibitors of PDE3 are used for the treatment of heart failure (milrinone), PDE4 inhibitors are used for treating inflammatory conditions such as COPD (roflumilast) and psoriatic arthritis (apremilast), and PDE5 inhibitors are used for erectile dysfunction (sildenafil, tadalafil, and vardenafil) and pulmonary hypertension (sildenafil). With the proven track record of identifying drugs that inhibit PDEs, there was significant excitement in the pharmaceutical world when PDE10A was identified as a potential new target in 1999 (Fujishige et al., 1999; Loughney et al., 1999; Soderling et al., 1999). PDE10A was found to be capable of hydrolyzing both cAMP and cGMP and mRNA for the enzyme was found to be highly localized to the brain and testes. Within the brain, mRNA expression is highest in the striatum and within this brain region expression is exclusive to striatal medium spiny neurons; high levels of protein expression also correspond with this restricted mRNA distribution pattern (Seeger et al., 2003; Xie et al., 2006). Thus, PDE10A is a unique signaling molecule in being highly expressed in only a single neuronal population and in having a singular molecular signaling role. This localization prompted an intensive effort to determine the role of PDE10A in regulating striatal function and to investigate the potential therapeutic utilities of PDE10A inhibitors (Kehler and Nielsen, 2011; Chappie and Verhoest, 2014; Charych and Brandon, 2014).

The striatum is a large nucleus comprised primarily of PDE10A-expressing medium spiny neurons (MSNs) that functions as the gateway for the input and processing of cortical information by the basal ganglia circuit (Albin et al., 1989; Haber, 2016). The MSNs are also recipient of a dense dopaminergic input from the substantia nigra and ventral tegmental area. In roughly half of the MSNs, the dopamine signal is transduced through dopamine D1 receptors and in the other half this signal is transduced through dopamine D2 receptors. The efferents of these two classes of MSNs delineate two parallel information processing streams, the direct and indirect striatal output pathways. These two pathways coordinate in the dynamic integration of cortical information with dopamine-coded reward/salience information to select advantageous behaviors while suppressing less advantageous options (Wichmann and DeLong, 1996). Dysfunction in this circuitry is implicated in a range of neuropsychiatric and neurodegenerative conditions (Graybiel, 2000). Notably, inhibition of dopamine D2 receptors on indirect pathway MSNs is putatively the mechanism of antipsychotic action of the D2 receptor antagonists, the standard of care pharmacotherapy for the treatment of psychosis in schizophrenia (Seeman, 2010; McCutcheon et al., 2019). Rodent behavioral studies in mice with genetic deletion of *PDE10A* (Siuciak et al., 2006b; Sano et al., 2008; Piccart et al., 2014) and mice or rats treated with PDE10A inhibitors such as papaverine (Siuciak et al., 2006a), PQ-10 (Chappie et al., 2007), TP-10 (Schmidt et al., 2008), THPP-1 (Smith et al., 2013), and JNJ-42314415 (Megens et al., 2014a) revealed that PDE10A inhibition causes behavioral effects similar to D2 antagonists. In fact, the similarities to D2 antagonists were considered very suggestive of the potential for antipsychotic activity, launching an industry-wide effort to develop PDE10A inhibitors as a new class of antipsychotic agents that regulate striatal function outside of the traditional neurotransmitter/receptor realm. Extensive reviews of the work to identify PDE10A inhibitors have been published (Chappie et al., 2012; Jørgensen et al., 2013; Jankowska et al., 2019). Recent searches have identified >150 PDE10A inhibitor patents with >15 companies represented. Ultimately, these efforts resulted in 12 reported clinical candidates and 4 clinically validated PDE10A PET ligands (Geerts et al., 2017).

In clinical studies to date, PDE10A inhibitors have generally been found to be safe and well-tolerated at doses yielding exposures in the range targeted for efficacy (Tsai et al., 2016). Significantly, PDE10A inhibitors were found to be psychoactive in the targeted exposure ranges, producing a state characterized as “awake sedation” or “conscious sedation,” as discussed at a NIMH-sponsored workshop on PDE10A held January 25, 2013 at the NIH Neuroscience Center in Rockville, MD, USA. At higher exposures, PDE10A inhibitors were found to induce sporadic dystonia, particularly of the tongue, head, and neck. This motor side effect is consistent with the compounds modulating basal ganglia circuitry, albeit in a maladaptive fashion.

Two companies, Pfizer and Takeda, have published results of Phase II efficacy studies with PDE10A inhibitors in patients experiencing acute psychosis associated with chronic schizophrenia. Pfizer's PF-02545920 was first characterized for PDE10A enzyme occupancy in healthy volunteers at doses of 10 mg and 20 mg using PET imaging (Delnomdedieu et al., 2017). PDE10A enzyme occupancy was demonstrated to be 14–27% following the 10 mg dose and 45–63% following the 20 mg dose. Both doses were safe and well-tolerated. PF-02545920 was then tested for antipsychotic efficacy in patients with schizophrenia experiencing an acute exacerbation of psychotic symptoms (Walling et al., 2019). The study involved 4 weeks of treatment in patients randomly assigned to receive either 5 mg or 15 mg of PF-02545920 (Q12H, 74 patients per treatment group). Comparator cohorts received placebo (74 patients) or 3 mg of risperidone (Q12H, 37 patients), a D2 antagonist that is a standard of care. Risperidone showed a statistically significant difference from placebo in alleviating symptoms based on the Positive and Negative Syndrome Scale (PANSS) total score at the end of 4 weeks. However, neither dose of PF-02545920 produced a statistical separation from placebo at any time point.

Pre-clinical data suggested that PDE10A inhibition may also augment the antipsychotic activity of D2 antagonists. To investigate this potential therapeutic utility, Pfizer conducted a second clinical study in schizophrenia patients receiving a D2 antagonist but whose symptoms were sub-optimally controlled (DeMartinis et al., 2019). The study involved 3 dose groups: PF-02545920 at 5 mg (Q12H, 78 patients) or 15 mg (Q12H, 82 patients), or placebo (80 patients) with treatment planned for 12 weeks. However, the study was halted due to an interim futility analysis indicating a low probability of any significant additional beneficial response when PF-02545920 was added to standard of care D2 antagonists.

Takeda developed a PDE10A inhibitor designated as TAK-063. Early clinical characterization using PET imaging indicated TAK-063 can be dosed to achieve PDE10A enzyme occupancies from 2.8 to 72.1% with good toleration (Takano et al., 2016). Takeda then conducted a clinical trial in schizophrenia patients experiencing an acute exacerbation of psychotic symptoms (Macek et al., 2019). The study involved the dosing of TAK-063 (20 mg; 83 patients) or placebo (81 patients) for 6 weeks. Modeling from the PET study indicated this 20 mg dose would yield ~30% PDE10A enzyme occupancy. Unfortunately, the study did not achieve its primary endpoint of a significant change from baseline in the PANSS score. However, Takeda noted that three secondary endpoints were improved in the TAK-063 group. Those endpoints were the Clinical Global Impression severity (CGI-S) scores, Clinical Global Impression improvement (CGI-I) scores, and the percentage of CGI-I responders.

The positive movements in the secondary endpoints of the TAK-063 study were also measured in the PF-02545920 monotherapy trial and were found to be non-significantly changed at either 5 or 15 mg. Importantly, the positive control in the study, risperidone, was found to have significant positive effects vs. these secondary endpoints suggesting that the study was capable of sensing changes to these measures over the duration of the study. There were also no effects of PF-02545920 on global clinical measures in the study in which this agent was added to D2 antagonist treatment. The enzyme occupancy levels for the 15 mg dose of PF-02545920 was in a similar range to that estimated for the 20 mg dose of TAK-063, suggesting that enzyme occupancy is not a factor in the difference in secondary outcomes measures. Durations of treatments were also in the same range, suggesting this factor also does not account for the difference.

As this review was in preparation, H. Lundbeck A/S announced halting a trial based on an interim futility analysis of the effects of their PDE10A inhibitor Lu AF11167 against persistent prominent negative symptoms in patients with schizophrenia (BNSS). Secondary endpoints in the Lu AF1167 trial were the PANSS and the study protocol called for the drug to be administered for 12 weeks. Although additional PDE10A inhibitors (Geerts et al., 2017) have advanced to early clinical safety studies, searches of company websites suggest that efforts regarding the PDE10A mechanism with respect to schizophrenia have been discontinued.

Pfizer also conducted a proof-of-concept Phase II study of the efficacy of PF-02545920 to improve symptoms in patients with Huntington's disease (Delnomdedieu et al., 2018). Doses of 5 or 20 mg were used in a study of over 200 early-stage symptomatic patients. There was no significant effect of treatment on the primary outcome measure, the Unified-Huntington's-Disease-Rating-Scale Total-Motor-Score (UHDRS-TMS). However, a dose-dependent improvement was observed on an exploratory measure, the Q-motor score, suggesting a possible effect of the drug on motor coordination.

In summary of the clinical trial data currently available, PDE10A inhibitors were demonstrated to be psychoactive in that they produced somnolence or sedation in all clinical studies publicly reported. However, the Pfizer or Takeda PDE10A inhibitors did not produce clinically meaningful improvements in positive symptoms in patients suffering schizophrenia as measured using the PANSS scale, the primary outcome measures in these studies. Based on the preliminary report from Lundbeck, there was apparently no robust effect of this mechanism on negative symptoms. In the Takeda study in schizophrenia patients exhibiting acute exacerbation of symptoms, there was evidence of a favorable change in global clinical impressions; however, this was not replicated in the Pfizer studies. Given the compelling preclinical data and biological rationale suggesting that PDE10A inhibition would positively impact schizophrenia, the clinical results from Pfizer, Takeda, and Lundbeck call for a reevaluation of our hypotheses regarding the mechanism(s) by which PDE10A inhibitors and D2 antagonists may ameliorate psychosis. There is now a wealth of data on the physiology of PDE10A and preclinical data on the effects of PDE10A inhibitors that can be compared to that of D2 receptors and D2 receptor antagonists. Our purpose here is to review and synthesize this extensive data set with an ultimate goal of understanding why these two mechanisms do not produce similar clinical activity and to highlight knowledge gaps that impede full interpretation of the clinical data. Understanding the apparent lack of predicted antipsychotic activity will hopefully inform future efforts to develop new antipsychotic therapies and justify/enable continued drug development research for this indication. We also hope this review may serve in the formulation of new hypotheses around therapeutic uses for PDE10A inhibitors. To set the stage, we first provide a brief review of the physiology of striatal MSNs and dopamine signaling within these neurons.

## Striatal MSNs–A Key Cellular Target of Antipsychotic Drugs

As used here, *striatum* refers to the contiguous subcortical nuclei of caudate n., n. accumbens, and olfactory tubercle (rodent nomenclature), the input loci of the cortico-striato-nigral-thalamic loop known as the basal ganglia circuit (Albin et al., 1989; Gerfen, 1992; Haber et al., 2000). The MSNs comprise the major neuronal type in the striatum—in rodents MSNs are estimated to comprise 90–95% of striatal neurons, whereas in humans the percentage is slightly lower. These GABAergic projection neurons receive an extensive, topographically organized, excitatory glutamatergic input from cortex and thalamus (Bolam et al., 2000; Haber et al., 2000; Haber, 2016). The MSNs are also the recipient of a topographically organized dopaminergic input from substantia nigra and ventral tegmentum (Bolam et al., 2000). There are two anatomically and biochemically defined subsets of MSNs (Alexander and Crutcher, 1990; Gertler et al., 2008). MSNs of the *direct* pathway express dopamine D1 receptors and the neuropeptides dynorphin and substance P. Direct pathway MSNs project *directly* to and inhibit the output nuclei of the basal ganglia, the substantia nigra/entopeduncular n., which in turn project to and inhibit the thalamus. Activation of direct pathway MSNs dis-inhibits the excitatory thalamic output to cortex. MSNs of the *indirect* pathway express dopamine D2 receptors, adenosine A2A receptors, and the neuropeptide enkephalin. These MSNs also modulate the activity of the substantia nigra/entopeduncular n., but in this case *indirectly* via a multi-synaptic pathway through the external globus pallidus and subthalamic n. with the end result being dis-inhibition of the output nuclei to suppress thalamic feedback to cortex. In the classical model of the basal ganglia circuit, the direct striatal output pathway broadly functions to facilitate behavioral responses, whereas the indirect striatal output pathway functions to suppress behavioral responses that compete with those being facilitated through the direct pathway (Alexander and Crutcher, 1990; Calabresi et al., 2014).

The excitatory glutamatergic drive on MSN activity is regulated by the peri-synaptic dopaminergic input arising from substantia nigra and ventral tegmental nucleus (Surmeier et al., 2007). The intracellular signaling triggered by dopamine in the MSN is multi-faceted (Valjent et al., 2019). The most well-studied mechanisms down-stream of dopamine receptor activation are G protein-dependent modulations of cAMP formation. D1 receptors are positively coupled to adenylate cyclase, whereas D2 receptors are negatively coupled to adenylate cyclase (Bibb, 2005). Thus, dopamine release in striatum causes an increase in cAMP in direct pathway neurons while inhibiting cAMP synthesis in indirect pathway neurons. D2 receptor antagonists increase cAMP in indirect pathway neurons by reducing the D2 receptor brake on cyclase activity. D1 receptor stimulation and D2 receptor inhibition also increase cGMP synthesis in striatum (West, 2016). Dopamine-regulated striatal cGMP synthesis is driven by nitric oxide (NO) stimulation of soluble guanylate cyclase, which is expressed by both direct and indirect pathway MSNs. However, NO is delivered by *inter-neuronal* diffusion following stimulation of neuronal nitric oxide synthesis (nNOS) located in a small population of nNOS-positive striatal interneurons. Thus, cGMP signaling may not be as discreetly segregated in direct and indirect pathway neurons as is cAMP signaling. Both D1 and D2 receptors also signal through an interaction with β-arrestin to regulate an Akt/GSK3b signaling cascade independently of G-protein signaling (Del'Guidice et al., 2011). Furthermore, dopamine receptors may form functional heteromeric complexes through dimerization with adenosine, metabotropic glutamate, peptidergic, or serotonin receptors (Perreault et al., 2014; Borroto-Escuela et al., 2020). These dopamine receptor heteromers have unique downstream signaling signatures in the MSNs. Additional mechanisms by which D2 antagonists modulate striatal information processing include effects on D2 receptors on glutamate terminals (Bamford et al., 2004) as well as on striatal interneurons (Centonze et al., 2003). Blockade of non-striatal D2 receptors may also contribute to the therapeutic mechanism of action of this class (O'Donnell, 2012).

It is an open question how the different D2 receptor signaling mechanisms outlined above are impacted by D2 receptor antagonists to mediate the changes in basal ganglia information processing that results in the suppression of psychosis in schizophrenia (Boyd and Mailman, 2012; Martel and Gatti McArthur, 2020). The molecular signaling mechanisms activated by PDE10A inhibitors intersect with those activated by D2 receptor antagonists at the level of cAMP and cGMP signaling. Significant to the perspective of this review, PDE10A inhibitors and dopamine D2 receptor antagonists have many similar effects on MSN activity and basal ganglia function downstream of the respective proximal molecular signaling mechanisms. It is these similarities that supported advancing the PDE10A inhibitors as potential antipsychotics (Menniti et al., 2007). Thus, the next section of this review will focus on the role of PDE10A in regulating cyclic nucleotide signaling and function in MSNs and a comparison of such effects to dopamine receptor modulators.

## PDE10A—A Phosphodiesterase Highly Enriched in Striatal MSNs

Discovery of the PDE10A gene in 1999 (Fujishige et al., 1999; Loughney et al., 1999; Soderling et al., 1999) resulted from a bioinformatics search for genes with homology to known PDEs, enabled by the newly available complete sequence of the human genome. While high levels of PDE10A mRNA were detected in brain and testes, high levels of PDE10A protein were detected only in brain (Coskran et al., 2006). Analyses of both PDE10A mRNA and protein expression revealed that the distribution of this phosphodiesterase is further delimited to high expression only in striatal medium spiny neurons (Xie et al., 2006). PDE10A is expressed as 3 major splice variants, PDE10A1, A2, and A3, although as many as 15 minor variants may also exist (Fujishige et al., 2000; MacMullen et al., 2017). Immunohistochemical analyses indicate PDE10A distributes throughout the MSNs, i.e., in soma and throughout the complete dendritic and axonal compartments (Seeger et al., 2003). In biochemical analyses of striatal tissue, which contains MSN cell bodies, dendrites, and axon collaterals, PDE10A is primarily membrane bound (Xie et al., 2006). Membrane localization appears to be the result of irreversible n-terminal palmitoylation (Charych et al., 2010). Electron microscopic analysis revealed the protein to distribute into dendritic spines, including juxtaposed to the post-synaptic density (Xie et al., 2006). Consistent with this observation, biochemical analyses indicate that the enzyme is incorporated into a post-synaptic complex that includes NMDA receptors, PSD95, AKAP150, and PKA (Russwurm et al., 2015). In contrast, there is no information on subcellular localization of PDE10A in MSN axons and terminals in globus pallidus and substantia nigra.

PDE10A mRNA and protein are detected in other neurons throughout the brain, albeit at levels much lower than in MSNs (Seeger et al., 2003; Coskran et al., 2006). In forebrain neurons outside of striatum, PDE10A-like immunoreactivity is confined to cell nuclei and/or the perinuclear compartment. Nuclear localization of PDE10A protein in hippocampus was confirmed in cell fractionation studies (Giralt et al., 2013). PDE10A mRNA levels are upregulated in hippocampus by the induction of LTP (O'Connor et al., 2004), and PDE10A mRNA and protein also vary with a diurnal rhythm in pineal gland (Spiwoks-Becker et al., 2011). Collectively, these data imply function roles for the enzyme in non-striatal brain regions. However, pharmacological inhibition of PDE10A causes no detectable changes in cyclic nucleotide levels or gene expression in non-striatal forebrain tissue (Kleiman et al., 2011), in contrast to robust changes in striatal tissue. Thus, PDE10A appears to have a unique role in regulation of striatal MSN function.

Striatal MSNs contain a high density of cAMP and cGMP signaling components, including the highest levels of phosphodiesterase in brain (Lakics et al., 2010; Kelly, 2014). PDE10A, highly expressed in both direct and indirect pathway MSNs, is anatomically placed to regulate the activity of both direct and indirect pathways. As a dual substrate phosphodiesterase, PDE10A is capable of regulating both cAMP and cGMP signaling in MSNs of both pathways. Next, we review what is known about the role of PDE10A in regulating cyclic nucleotide signaling in MSNs.

## PDE10A Regulation of Cyclic Nucleotide Signaling in MSNs

Initial characterizations of PDE10A demonstrated that the enzyme hydrolyzes both cAMP and cGMP (Fujishige et al., 1999; Soderling et al., 1999). The affinity of recombinant PDE10A for cAMP is considerably greater than for cGMP, and it was suggested that the enzyme may be a cAMP-regulated cGMP-ase. Subsequent studies of the effects of PDE10A inhibitors on striatal cyclic nucleotide levels in rodents reviewed below indicated that PDE10A, in fact, regulates both cAMP and cGMP signaling in striatum. However, there is no evidence for competitive substrate interactions; i.e., the regulation by PDE10A of cAMP signaling is independent of the regulation of cGMP signaling and *vice versa*.

Systemic administration of PDE10A inhibitors to mice causes a robust increase in striatal cGMP levels relative to levels in the absence of inhibitor (i.e., “basal” levels)^1^. The increase in cGMP levels may be 5-fold or higher than basal levels (Chappie et al., 2007; Schmidt et al., 2008; Malamas et al., 2011; Suzuki et al., 2015). An increase in cAMP over basal level is also observed in striatum (Schmidt et al., 2008; Malamas et al., 2011; Suzuki et al., 2015). The magnitude of the cAMP increases in absolute terms is similar to that for cGMP. However, basal levels of cAMP are about 10-fold higher than for cGMP and so the effect of PDE10A inhibition on cAMP levels as a ratio to basal levels are modest and more difficult to reliably detect. Systemic administration of PDE10A inhibitors also produces robust increases in both cAMP and cGMP levels in rat striatum^2^. These data indicate that PDE10A regulates actively turning over pools of cGMP and cAMP in striatal MSNs, even in the absence of any overt behavioral or pharmacological stimulus to drive cyclic nucleotide synthesis.

### cGMP Signaling

The pool of cGMP regulated by PDE10A is derived from soluble guanylate cyclase (sGC) stimulated by nitric oxide (NO) synthesized by neuronal nitric oxide synthase (NOS) (Threlfell et al., 2009; Padovan-Neto et al., 2015). Striatal MSNs express high levels of sGC, an enzyme that, when activated by NO, catalyzes the cyclization of GTP to form cGMP. NO is a diffusible intra- as well as inter-cellular second messenger formed from L-arginine by three different synthases, neuronal NOS, endothelial NOS, or inducible NOS. In striatum of mice with genetic deletion of nNOS, basal cGMP is decreased 80–90%. Furthermore, the effect of PDE10A inhibition to increase cGMP is completely abrogated (Padovan-Neto et al., 2015). Similar effects on both basal and PDE10A inhibitor enhanced cGMP levels are observed after systemic administration of NOS or nNOS-selective inhibitors. Genetic deletion of nNOS or administration of nNOS inhibitors also completely block the increase in cGMP caused by D2 antagonists or dopamine D1 receptor agonists (West, 2016). The locus of nNOS driving this striatal cGMP synthesis is nNOS-positive striatal interneurons. In addition to expressing high levels of nNOS, these interneurons express the neuropeptides somatostatin and NPY, and are characterized electrophysiologically as having a low threshold for induction of Ca^2+^ spikes and for sustaining a prolonged depolarized membrane potential (Kawaguchi, 1993). They are referred to in the literature as SOM+ or PLTS striatal interneurons.

**Figure 2:**
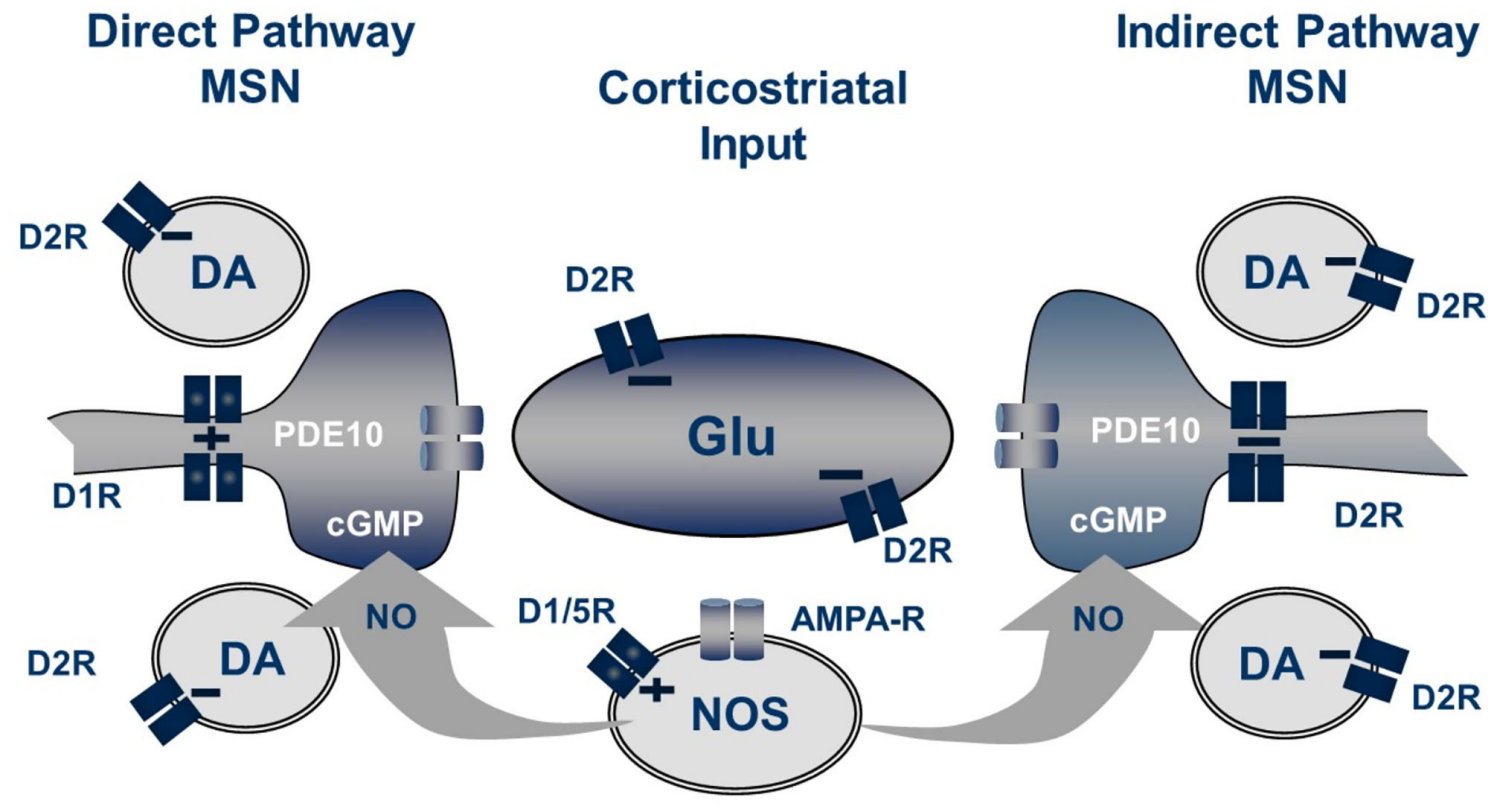
Striatal regulation of cGMP signaling in direct and indirect pathway MSNs. The generation of NO from NOS+ interneurons is dependent upon both D1-like receptors and glutamate acting on AMPA receptors while the concentration of cGMP in MSN is regulated by phosphodiesterase activities including PDE10A. Not shown are the cholinergic interneurons which also play a role in this regulation.

Striatal nNOS positive interneurons integrate a variety of synaptic inputs, including glutamatergic input from cortex, cholinergic input from striatal fast-spiking interneurons and GABAergic inputs from striatal and extra-striatal sources (Tepper et al., 2010). Notably, a principal driver for cell firing is activation of dopamine D1-like (probably D5) receptors expressed by these cells (Centonze et al., 2002). Consistent with this scheme, we observe that systemic administration of the D1 agonist SKF-81297 causes a modest increase in striatal cGMP level, whereas the AMPA receptor antagonist CP-465,022 caused a reduction. However, the D1 antagonist SCH-23390 had no effect on the PDE10A inhibitor-induced increase in striatal cGMP. Furthermore, the increase in striatal cGMP levels caused by a PDE10A inhibitor administered with a D1 agonist were found to be additive, not synergistic. Surprisingly, the D2 antagonist haloperidol was found to cause a more robust increase in striatal cGMP than the D1 agonist, despite the fact that the nNOS positive interneurons do not express D2 receptors (Centonze et al., 2002; Tepper et al., 2010). Furthermore, the D2 agonist quinpirole, while having no effect on cGMP levels when administered alone, attenuated the PDE10A inhibitor induced cGMP increase. Conversely, the increase in cGMP levels caused by PDE10A and D2 inhibition are super-additive. Thus, activation of the nNOS-positive interneurons results in the formation of NO, which diffuses into MSNs to stimulate the formation of cGMP by sGC. Whereas, NO-driven cGMP synthesis is the source of the PDE10A regulated cGMP pool, there appears to be compartmentalization and differential regulation of cGMP pools regulated by D1 and D2 receptor stimulation. Specifically, PDE10A appears to regulate a cGMP pool linked to D2 receptor activity but does not directly regulate the cGMP pool downstream of D1 receptor activation (Figure 2).

### cAMP Signaling

Systemic administration of PDE10A inhibitors to mice or rats increase striatal cAMP levels (Schmidt et al., 2008; Suzuki et al., 2015). However, as noted above, this effect of the inhibitors is difficult to quantify against the background levels of cAMP, which in turn makes it difficult to study the nature of the upstream signaling mechanisms driving the cAMP pool(s) regulated by PDE10A. To overcome this obstacle, we studied change in the level of CREB phosphorylation as a surrogate for change in cAMP. Systemic administration of PDE10A inhibitors to mice results in a rapid and robust increase in striatal levels of phospho-CREB (Schmidt et al., 2008; Smith et al., 2013; Suzuki et al., 2015). The phospho-CREB response to PDE10A inhibition is downstream of cAMP signaling since it is not affected by genetic deletion of nNOS, which completely eliminates the PDE10A inhibitor-induced increase in striatal cGMP (see above). Furthermore, for TP-10, the dose-response relationship for increasing phospho-CREB paralleled that for the increase in cAMP and increases in phospho-CREB were temporally aligned with plasma drug concentrations (Schmidt et al., 2008). Pharmacological inhibition of dopamine D1 receptors attenuated the increase in phospho-CREB caused by PDE10A inhibition. However, the effect of a dopamine D1 receptor agonist to increase phospho-CREB levels was additive with that of a PDE10A inhibitor, not synergistic as would be predicted if PDE10A was the principal phosphodiesterase regulating the D1 receptor stimulated cAMP pool driving CREB phosphorylation. Stimulation of D2 receptors, which are negatively coupled to adenylyl cyclase, attenuated the PDE10A inhibitor-induced increase in phospho-CREB. Again, however, the effect of PDE10A inhibition and D2 receptor inhibition were additive and not synergistic. Thus, changes in phospho-CREB, as a surrogate for changes in cAMP, indicate that PDE10A plays a role in regulating the cAMP signaling pools downstream of both dopamine D1 and D2 receptor signaling. However, the lack of synergistic effects of PDE10A inhibition with either a D1 agonist or D2 antagonist indicate that PDE10A is but one of several regulatory factors and one of several cAMP phosphodiesterases highly expressed in striatum (Polito et al., 2013).

Indirect pathway MSNs express adenosine A2 (A2A) receptors positively coupled to adenylyl cyclase. Nishi et al. (2008) reported that the PDE10A inhibitor papaverine increased phosphorylation of DARPP-32 at the Thr34 (PKA) site in mouse brain slices containing striatum. This effect of papaverine was potentiated by the A2A agonist CGS21680 and partially inhibited by A2A antagonist ZM241385. These data indicate that PDE10A may play a role in regulating cAMP signaling driven by A2A receptor activation in indirect pathway MSNs. Adenylyl cyclase activity in striatum is also regulated by calcium signaling mechanisms including those triggered by ionotropic glutamate receptor activation. However, we found that systemic administration of NMDA or AMPA receptor antagonists did not attenuate the increase in phospho-CREB induced by administration of a PDE10A inhibitor. Thus, the complex pharmacology of PDE10A inhibitors on cAMP levels likely reflect the complexity and compartmentalization of cAMP signaling in striatum and highlight the limitations of using bulk tissue measurements of signaling molecules to investigate such compartmentalized systems.

Finally, we note that there is virtually no data on the cyclic nucleotide signaling cascades regulated by PDE10A in the axons and terminals of MSNs, where PDE10A is also highly expressed.

## Consequences of PDE10A Inhibition Downstream of Striatal Cyclic Nucleotides

Consistent with localization of the enzyme in both MSN populations, PDE10A inhibition alters cyclic nucleotide signaling activated by D1 and D2 receptors. There are both qualitative and quantitative differences in these effects. Significant to this discussion, the consequences of PDE10A inhibition appear to be biased for greater activation of indirect pathway MSNs, at least in rodent systems.

Studies by Nishi et al. (2008) in brain slices containing striatum first indicated that PDE10A inhibition has an effect biased toward activation of indirect pathway MSNs. In studies of protein phosphorylation in striatal slices from mouse, Nishi et al. reported that PDE10A inhibition increased phosphorylation of DARRP-32 at Thr34, the AMPA receptor subunit GluR1 at Ser 845, and ERK2 at Thr202/Tyr204. Effects of PDE10A inhibition on DARPP-32 phosphorylation were not affected by inhibition of soluble guanylyl cyclase with ODQ, indicating DARPP-32 phosphorylation is downstream of cAMP and PKA signaling. DARPP-32 phosphorylation in direct and indirect pathway MSNs were further analyzed in slices from mice in which DARRP-32 was differentially tagged with Flag or Myc, respectively. PDE10A inhibition increased phosphorylation of DARRP-32 pulled down with either tag, consistent with effects of PDE10A inhibition on cAMP signaling in both MSN populations. However, the efficacy of PDE10A inhibition to increase DARPP-32 phosphorylation was greater for Myc-tagged protein, i.e., that pulled down from indirect pathway MSNs. Nishi et al. concluded that PDE10A inhibition has a greater impact on signaling in MSNs of the indirect pathway and highlighted that the effects of PDE10A inhibition bore resemblance to those of D2 antagonists.

A similar conclusion was reached by Vincent and colleagues, in this case examining the effects of PDE10A inhibition in striatal slices using cAMP or PKA biosensors (Polito et al., 2015). Biosensor responses in direct and indirect pathway MSNs were distinguished pharmacologically based on MSN responsiveness to dopamine D1 or adenosine A2A agonists, respectively. In slices transfected with the cAMP biosensor Epac-SH150, PDE10A inhibition equivalently increased biosensor signal in both direct and indirect pathway MSNs. In contrast, in slices transfected with PKA biosensor AKAR3, PDE10A inhibition resulted in increased biosensor signal in indirect pathway MSNs but not in direct pathway MSNs. The biased activation of PKA-signaling in indirect pathway MSNs was also observed *in vivo* in mice treated with a PDE10A inhibitor. These studies used mice in which indirect pathway MSNs were identified by expression of EGFP-tagged dopamine D2 receptors. PDE10A administration increased PKA-dependent histone H3 phosphorylation exclusively in EGFP-positive MSNs. Thus, while PDE10A regulates cAMP signaling in MSNs of both the direct and indirect pathway, the downstream consequences are of greater impact in indirect pathway MSNs.

A differential effect of PDE10A inhibition on indirect pathway MSNs is also evident with regard to cGMP signaling, based on *in vivo* electrophysiological studies of PDE10A inhibitors on the excitability of MSNs by West and colleagues (Threlfell et al., 2009; Padovan-Neto et al., 2015). For these studies, MSNs of direct or indirect pathways were identified by whether or not, respectively, they were activated by antidromic stimulation from substantia nigra, the terminal zone for direct pathway MSNs. Strikingly, PDE10A inhibition increased excitability of indirect pathway MSNs to cortical stimulation without effecting excitability of direct pathway MSNs. This effect of PDE10A inhibition was abrogated in nNOS knock out mice, indicating mediation by cGMP signaling (Padovan-Neto et al., 2015). A subtle effect of PDE10A inhibitors on direct pathway MSNs was observed by Threlfell et al.; the number of MSNs activated by antidromic stimulation of substantia nigra was increased in animals treated with a PDE10A inhibitor (Threlfell et al., 2009). This implied that PDE10A inhibition increased axonal excitability of direct pathway MSNs. However, whether this effect was abrogated in the nNOS knock out mice was not tested so it is not known whether this effect was mediated by cGMP. A similar analysis of axonal excitability of indirect pathway MSNs was not technically feasible. Thus, PDE10A regulates cGMP signaling in MSNs of the indirect pathway with consequences biased toward activation of indirect pathway MSNs. Given that NO is a diffusible messenger, we may conjecture that PDE10A also regulates cGMP signaling in direct pathway MSNs, but downstream effectors of such signaling have not been established.

PDE10A plays a significant role in regulation of gene expression changes in MSNs. Strick et al. found that PDE10A inhibition led to increases in expression of both substance P and enkephalin mRNA in striatum (Strick et al., 2010); see also (Suzuki et al., 2015). Since these markers are expressed selectively by direct and indirect pathway MSNs, respectively, it was concluded that PDE10A regulates gene expression in both MSN populations. This conclusion was supported in a more recent study in which TAK-063 was found to induce increases in striatal cfos expression in both direct and indirect pathway MSNs (Nakatani et al., 2017). In the earlier Strick et al. study, the PDE10A inhibitor-induced increase in cfos expression was unaffected by genetic deletion of nNOS, indicating the cfos response is downstream of cAMP signaling. Microarray profiling of mRNA expression indicates that the number of genes under regulation by PDE10A is quite substantial and restricted to striatum (Kleiman et al., 2011). Thus, PDE10A functions as a brake on a complex transcriptional program in direct and indirect pathway MSNs, indicating that inhibitors of the enzyme may have long term consequences to the function of these neurons.

## Behavioral Effects of PDE10A Inhibition in Rodents

Given the prominent localization of PDE10A to striatal medium spiny neurons, the effects of PDE10A inhibition have been studied in rodent behavioral paradigms that are sensitive to pharmacological manipulation of basal ganglia activity. In many cases, the effects of the PDE10A inhibitors were compared to antipsychotic dopamine D2 receptor antagonists and in some paradigms the effects of these two classes of compounds were very similar. This similarity is consistent with the physiological data reviewed above indicating a biased efficacy of PDE10A inhibition for activation of indirect pathway MSNs, i.e., those expressing dopamine D2 receptors. These observations served as a significant part of the rationale for advancing PDE10A inhibitors into clinical trials for the treatment of psychosis in schizophrenia. However, clear distinctions between these two classes of compounds have also been noted. These distinctions take on new significance in light of the lack of antipsychotic efficacy reported for PF-02545920, TAK-063 and Lu AF11167.

The most robust effects of PDE10A inhibition in rodents are inhibition of NMDA receptor channel blocker-induced hyperlocomotor activity and inhibition of conditioned avoidance responding. NMDA receptor channel blockers, including phencyclidine, ketamine, and MK-801, cause a spectrum of behavioral effects in humans that are similar to those experienced by patients with schizophrenia (Luby et al., 1959; Lahti et al., 2001). In fact, these drug effects in humans are the foundation for the hypothesis that *NMDA receptor hypofunction* is a primary mechanism underlying the expression of schizophrenia symptoms (Krystal et al., 2003; Javitt et al., 2012). In rodents, this class of compounds induce hyperlocomotor activity, among other behavioral effects. Thus, the ability of pharmacological agents to attenuate NMDA channel blocker-induced hyperlocomotor activity is considered indicative of potential for clinical antipsychotic activity (Jentsch and Roth, 1999). PDE10A inhibitors very effectively block such hyperlocomotor activity—effects are dose dependent and inhibition may be complete (Siuciak et al., 2006a; Chappie et al., 2007; Schmidt et al., 2008; Grauer et al., 2009; Malamas et al., 2011; Smith et al., 2013; Megens et al., 2014a; Suzuki et al., 2015). Furthermore, there is a close correspondence between the dose response of PDE10A inhibitors for inhibition of channel blocker-induced locomotor activity and increases in striatal cGMP levels.

It is hypothesized that aberrant dopamine signaling gives rise to the mis-attribution of stimulus salience, leading to the development of psychotic and delusional symptoms in schizophrenia (Kapur et al., 2005; Winton-Brown et al., 2014). The ability of D2 antagonists to reduce stimulus salience is hypothesized to underly the antipsychotic activity of this class, at least in part. Conditioned avoidance responding is a behavioral assay of stimulus salience and D2 antagonists are effective at inhibiting this behavior in rodents (Wadenberg, 2010). PDE10A inhibitors are also highly efficacious at blocking conditioned avoidance responding (Schmidt et al., 2008; Grauer et al., 2009; Malamas et al., 2011; Smith et al., 2013; Suzuki et al., 2015). The dose response for this effect overlays with that for inhibition of channel blocker-induced hyperlocomotor activity and increasing striatal cyclic nucleotides. Thus, the effectiveness and tight PK/PD relationship for PDE10A inhibitors to block NMDA receptor channel blocker-induced hyperlocomotion and conditioned avoidance responding were foundations of the rationale for investigating this class as antipsychotic agents.

PDE10A inhibitors bear similarity to D2 antagonists in several other assays. PDE10A inhibitors ameliorate apomorphine-induced agitation in rats (Megens et al., 2014b) and deficits in extradimensional set shifting caused by subchronic NMDA antagonist administration in rats (Rodefer et al., 2005; Shiraishi et al., 2016). PDE10A inhibitors also block amphetamine-stimulated locomotor activity, although less effectively than for inhibition of NMDA channel blocker-induced activity (Siuciak et al., 2006a; Schmidt et al., 2008). In an empirical assay phenotyping drug-induced behavior in mouse, the SmartCube™, PDE10A inhibitors were identified as producing an antipsychotic-like profile similar to D2 antagonists (Roberds et al., 2011).

In contrast to the findings outlined above, PDE10A inhibitors lack efficacy in some rodent behavioral assays in which D2 antagonists are effective. These notably include induction of catalepsy and reversal of deficits in prepulse inhibition of startle. The available data suggest that the mechanism for the differences is that activation of direct pathway MSNs by PDE10A inhibitors counters the activation of indirect pathway MSNs underlying the behavioral responses.

Catalepsy in rodents is considered an indicator of liability to produce extrapyramidal side effects by agents that suppress psychosis (Hoffman and Donovan, 1995). Dopamine D2 antagonists produce a robust cataleptic response that monotonically increases with dose and time. This effect is attributable to activation of indirect pathway MSNs, which suppresses the behavioral response of stepping down from an elevated bar without impairing motor function (i.e., the ability to step down). In contrast, PDE10A inhibitors produce relatively little catalepsy (Schmidt et al., 2008; Grauer et al., 2009; Suzuki et al., 2015). In our experience, the cataleptic response to PDE10A inhibition was variable with both dose and time as well as with respect to replication with different compounds under nominally identical experimental conditions (Schmidt et al., 2008). Megens et al. (2014b) found that, whereas low doses of PDE10A inhibitors had no or limited propensity to induce catalepsy when administered alone, these compounds had potent and efficacious cataleptic effects when co-administration with a dopamine D1 receptor antagonist. This group also observed that such cataleptic effects were reversed at high doses of PDE10A inhibitors, which also inhibited the cataleptic effects of D2 antagonists. These data are consistent with a hypothesis that the weak and variable cataleptic effects of PDE10A inhibitors are due to direct pathway MSN activation, which counteracts the catalepsy-producing activation of the indirect pathway MSNs.

A similar scenario appears at play with regard to the effects of PDE10A inhibitors on prepulse inhibition of startle (PPI) in rat and mouse. PPI is a translatable experimental measure of sensorimotor gating (Swerdlow et al., 2008). PPI is deficient in schizophrenia as well as in other neuropsychiatric conditions and PPI deficits can be induced in rodents by manipulations of glutamatergic and dopaminergic neurotransmission that are used to model putative neurochemical abnormalities in schizophrenia (Geyer et al., 2001). D2 receptor antagonists ameliorate PPI deficits in rodents. Consequently, similar activity by new pharmacological agents may form part of the rationale for advancing such agents into clinical trials to test for antipsychotic efficacy. However, in the case of PDE10A inhibitors, inhibition of PPI is inconsistent, with some investigators reporting inhibition (Grauer et al., 2009; Das et al., 2014; Suzuki et al., 2016) and others reporting no activity (Schmidt et al., 2008; Weber et al., 2009; Suzuki et al., 2016), including for the same compound (i.e., TP-10). This discrepant effect of PDE10A inhibitors on PPI appears to stem from the competing activation of direct and indirect pathway MSNs. Gresack et al. (2014) reported that PDE10A inhibitors were effective at reversing PPI deficits induced by a dopamine D2 antagonist, quinpirole, but not by the mixed dopamine agonist apomorphine. However, PDE10A inhibitors were effective against apomorphine-induced deficits if co-administered with a dopamine D1 receptor antagonist. It was concluded that the activation of direct pathway MSNs attenuates effects on PPI that derive primarily from activation of indirect pathway MSNs.

A similar conclusion was proffered by Suzuki et al. in a study comparing the effects of TAK-063 and PF-02545920 on PPI among other assays (Suzuki et al., 2016). The Takeda group found that these two compounds were representative of two sub-classes of PDE10A inhibitors. TAK-063 represented a class with relatively faster enzyme dissociation rate than a class represented by PF-02545920. Significantly, the fast-dissociating compounds had a greater impact on indirect pathway activation relative to the direct pathway, whereas the slow dissociating class had a relatively more balanced activation of the two pathways. This difference was manifest as an ability of TAK-063 to ameliorate PPI deficits, whereas PF-02545920 was ineffective.

In summary of the above, the behavioral effects of PDE10A inhibitors in rodents reflects a unique pharmacology. In some part, PDE10A inhibitors bear resemblance to dopamine D2 receptor antagonists. This can be rationalized from the effects of PDE10A inhibitors on signaling in striatal MSNs, specifically, the apparent preferential effect of PDE10A inhibitors for activation of indirect pathway MSNs. Nonetheless, for some behaviors, PDE10A inhibitors lack the efficacy of D2 antagonists. This appears due to the effect of PDE10A inhibitors to activate direct pathway MSNs, which counters indirect pathway activation. This hypothesis is strengthened by the intriguing observations of Suzuki and Kimura of Takeda on differentiation of the PDE10A inhibitors based on enzyme off rate kinetics (Suzuki et al., 2016). These data clearly indicate that the unique behavioral profiles of PDE10A inhibitors reflects the balance of activity on direct and indirect pathway MSNs.

## Behavioral Effects of PDE10A Inhibition Non-human Primates

While rodent studies clearly reveal a unique pharmacology for PDE10A inhibitors that results from activation of both direct and indirect pathway MSNs, it is speculative as to how this pharmacology might translate to effects on human behavior. In this regard, the behavioral repertoire of non-human primates is obviously more comparable to that of humans. A study by Papa and collaborators in rhesus monkeys (Uthayathas et al., 2014) provides significant insight into the consequences PDE10A inhibition may have in humans and how this may differ from the consequences of D2 receptor inhibition.

The behavioral effects of the PDE10A inhibitor MP-10 (PF-02545920) were compared with that of the D2 antagonist risperidone in rhesus monkey. Doses of both compounds were chosen to mimic exposure ranges relevant to use in humans in clinical trials and clinical practice, respectively. Plasma exposures were verified and pharmacodynamic effects in brain were established by PET imaging of [^18^F]fluorodeoxyglucose uptake. A within subject design was employed in which each of 4 animals received each dose of MP-10 and risperidone on multiple occasions during which behavior was videotaped. Behavioral changes were scored using a standardized motor disability scale for parkinsonian primates and a newly designed “Drug Effects on Nervous System” scale to assess non-motor effects (Uthayathas et al., 2013). Each scale rated and assigned a score to a series of defined behaviors. Essentially, animals under the influence of these drugs underwent a careful neurological examination similar to what might be given in humans.

Overall, behavioral scores were similar for MP-10 and risperidone, at the level of both summary scores and scores on individually rated tests. However, subtle differences were noted that indicate a critical differentiation of the two compounds. The effects of risperidone were tightly dose responsive and reproducible in individual animals upon repeated exposures. In contrast, the effects of MP-10 were more all-or-none and there was notable variability in response of individuals from test session to test session. This variability was not accounted for by variability in exposures. Thus, both compounds produced qualitatively similar effects across a range of behaviors, but with a difference in dose responsiveness. It was hypothesized that the variable and all-or-none response pattern observed with MP-10 may reflect a “tipping point” in the activities of the direct and indirect pathways. Balanced activation of the two pathways with PDE10A inhibition results in little or no behavioral effect. However, tipping the balance toward indirect pathway activation results in a behavioral response similar to the D2 antagonist risperidone. The variability in response to MP-10 within individual animals is interpreted to indicate that this tipping point is relatively “sharp” and subject to subtle environmental and homeostatic influences that vary across nominally identical test sessions. It is also important to note that there were no emergent behavioral effects of MP-10 that might indicate a balance tipped toward direct pathway activation.

A more significant insight comes from the results of two other tests, the Kluver Board Test and Perch Test. In the Kluver Board Test, animals are required to reach into the openings of a plexiglass box with one finger to retrieve a reward. The difficulty of the task on individual trials is manipulated by varying the size of the opening. The numerical scores on the Kluver Board Test were identical for MP-10 and risperidone—at low doses animals made few errors whereas at high doses animals repeatedly failed to retrieve the reward. Significantly, the reason for the failures were different for the two compounds. Whereas, under risperidone the animals attempted to retrieve the reward but lacked the dexterity. In contrast, under MP-10 the animals stopped attempting to retrieve the reward. Results from the Perch Test further reflects this dichotomy. In this test, animals are required to scale a rod with perches to retrieve a reward at the top of the test enclosure. Under MP-10, animals showed no disability, whereas under risperidone animals lacked the coordination and balance to perform the task. Thus, these data indicate effects of PDE10A inhibition on motivational aspects of primate behavior that differs from that of D2 receptor inhibition, which is more highly related to motor fluency. As stated by Papa and colleagues—“MP-10- treated animals retained the ability to respond but did not engage tasks, whereas risperidone-treated animals retained the motivation to respond but were unable to perform the intended actions.”

## Discussion

At present, dopamine D2 receptor inhibition is the only well-proven pharmacology to ameliorate psychosis and delusions in patients with schizophrenia. Dopamine D2 receptors are densely expressed by MSNs of the indirect striatal output pathway. As noted earlier, D2 receptors are deployed in other striatal elements, such as on corticostriatal glutamate terminals and are expressed outside of the striatum. Notwithstanding, inhibition of D2 receptors on indirect pathway MSNs is a principal mechanism of their antipsychotic action. D2 receptor signaling in MSNs is complex. The most well-studied is G-protein mediated signaling to suppress adenylyl cyclase activity in response to dopamine. Thus, D2 antagonists disinhibit cAMP signaling in these neurons. D2 antagonists also increase cGMP signaling in MSNs. Augmentation of glutamatergic signaling likely plays a role in this effect although the coupling mechanisms are not understood in detail. In addition, D2 receptors signal through the β-arrestin/AKT/GSK3β kinase cascade, independently of G-protein-coupling. Further complexity derives from the dimerization of D2 receptors with a variety of other 7-transmembrane receptors that likely have unique signaling roles in the MSNs. The cumulative effect of D2 antagonists on these different signaling cascades is to increase the activity of indirect pathway MSNs and thereby bias striatal output toward the indirect pathway over the direct pathway. It is hypothesized that this biased activation of the indirect pathway suppresses the expression of psychotic symptoms. It is this hypothesis that framed the interest in PDE10A inhibitors as novel antipsychotics.

PDE10A is densely expressed by striatal MSNs and PDE10A inhibition increases both cAMP and cGMP signaling in these neurons. While PDE10A is expressed by both direct and indirect pathways MSNs, the net effect of PDE10A inhibition can be interpreted as a preferential activation of indirect pathway MSNs, based on biochemical and electrophysiological data. In this regard, PDE10A inhibitors bear similarity to D2 receptor antagonists and this suggested that PDE10A inhibitors similarly may be antipsychotic. The cap to this hypothesis was the finding that PDE10A inhibitors are highly efficacious for inhibiting conditioned avoidance responding in rodents, a behavioral assay of stimulus salience and an activity thought to be highly predictive of antipsychotic efficacy. The preclinical data with PDE10A inhibitors is summarized in Table 1. Nonetheless, PDE10A inhibitors from Pfizer, Takeda, and Lundbeck failed to exhibit robust antipsychotic efficacy in Phase II clinical studies. While the lack of clinical efficacy is disappointing, it affords a new opportunity to gain insights into the nature of antipsychotic drug action. Given the notable similarities in the effects of PDE10A inhibitors and D2 receptor antagonists across a range of experimental paradigms, why are only the latter compounds efficacious for ameliorating psychosis and delusions? As a first step toward answering this question, we re-visit key tenets supporting the rationale for investigating PDE10A inhibitors as antipsychotics and offer some reinterpretations of the supporting preclinical data. We then discuss some of the gaps in our knowledge that bear further investigation.

**Table 1:**
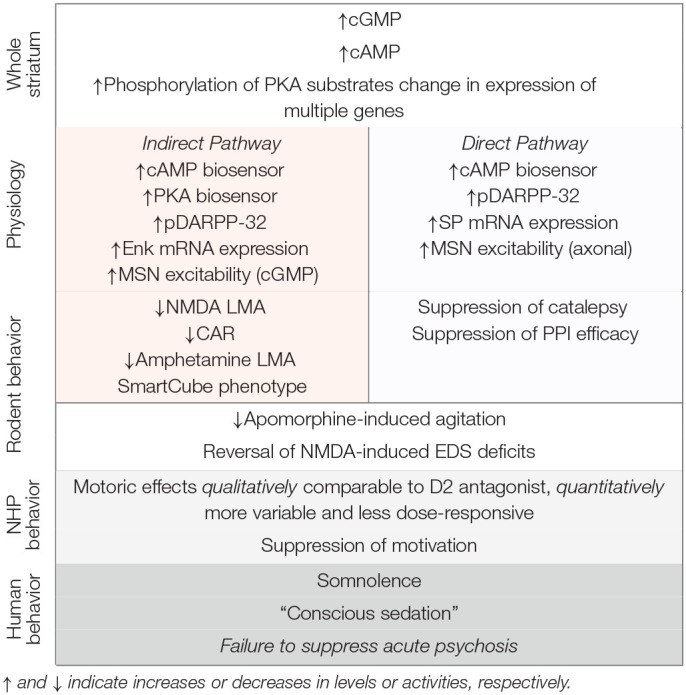
Summary of the effects of PDE10A inhibitors.

*↑ and ↓ indicate increases or decreases in levels or activities, respectively*.

At the molecular signaling level, the nominal intersection of PDE10A inhibitors and D2 antagonists is that both classes of compounds increase cAMP and cGMP levels in indirect pathway MSNs. However, based on the wealth of data reviewed above, it can be concluded that PDE10A is not directly coupled to D2 receptor cyclic nucleotide signaling. Instead, the pools of cyclic nucleotides impacted by these two pharmacologies overlap but are not synonymous^3^. Thus, in so far as D2 receptor antagonist modulation of cyclic nucleotide signaling is a “first molecular step” toward antipsychotic activity, then PDE10A inhibitors bear similarity to D2 antagonists but do not precisely activate the same signaling pools. Furthermore, D2 antagonists also impact D2 receptor signaling via the β-arrestin/AKT/GSK3β kinase cascade and by D2 receptor heteromers. There is emerging research suggesting that the modulation of these signaling pathways significantly contribute to antipsychotic activity (Del'Guidice et al., 2011; Borroto-Escuela et al., 2016, 2020; Weiwer et al., 2018). There are no documented linkages between PDE10A and these other D2 signaling mechanisms and so in this respect PDE10A inhibitors and D2 antagonists may be even further divergent.

The differences in molecular signaling notwithstanding, there is clear evidence from electrophysiological studies that both D2 antagonists and PDE10A inhibitors increase the activation of indirect pathway MSNs. A key tenet with regard to antipsychotic activity is that activation of indirect pathway MSNs is preferential to the activation of direct pathway MSNs. Such indirect pathway bias has a clear basis for D2 antagonists, given that D2 receptors are restricted to MSNs of this pathway. PDE10A inhibitors also have a greater impact on electrophysiological and biochemical measures in indirect pathway MSNs compared to direct pathway counterparts. Nonetheless, biochemical (Nishi et al., 2008; Strick et al., 2010; Polito et al., 2015) and behavioral (Gresack et al., 2014; Megens et al., 2014b) studies indicate that PDE10A inhibitors also impact direct pathway activity. The rodent behavioral studies of Gresack et al. (2014) on pre-pulse inhibition and Megens et al. (2014b) on catalepsy indicate that the direct pathway activation is consequential. Direct and indirect pathway MSNs are not a uniform neuronal population, beyond the well-recognized differences in dopamine signaling and neuropeptide expression. The two MSN populations have intrinsic differences in excitability, attributed to differences in the morphology of their dendritic trees (Gertler et al., 2008). The cortical inputs to the two MSNs populations also differ, arising from different layer 5 pyramidal neurons and the excitatory synapses formed with the respective MSN subtypes are morphologically and functionally distinct (Reiner et al., 2010). In this context, we raise the possibility that the differences in the effects of PDE10A inhibition on direct and indirect pathway MSNs may be more reflective of intrinsic differences in these two neuronal populations rather than a differential impact of PDE10A inhibition *per se*. Stated another way, biochemical and electrophysiological measures used so far may be overestimating the relative impact of PDE10A inhibition on indirect vs. direct pathway MSN activity, which is more balanced at the wholistic level of behavioral integration. This interpretation is subtle but important in that it further contrasts PDE10A inhibitors and D2 receptor antagonists.

The above discussion is germane to the question of whether increasing the bias of PDE10A inhibition toward indirect pathway activation will yield antipsychotic activity. This question is raised by Suzuki et al. (2016) with their findings regarding differences between TAK-063 and PF-02545920 in relative effects on direct and indirect pathway MSNs. The Takeda group found that these compounds were representative of sub-classes of PDE10A inhibitors differentiated based on enzyme dissociation rate. TAK-063, a fast-dissociating compound, had a greater impact on indirect pathway activation and this difference was manifest behaviorally as an ability of TAK-063 to ameliorate PPI deficits where PF-02545920 was ineffective. This difference becomes intriguing in light of the clinical findings in schizophrenia patients experiencing acute exacerbation of symptoms, where TAK-063 evidenced some efficacy on measures of global clinical impressions (Macek et al., 2019) but PF-02545920 did not have such effects (Walling et al., 2019, see above). This finding suggests that greater biasing PDE10A inhibition toward indirect pathway activation is a potential path toward more robust antipsychotic efficacy. Possibly countering this argument is the fact that there was no efficacy of PF-02545920 when administered with D2 antagonists (DeMartinis et al., 2019), a manipulation that would be expected to yield significant indirect pathway bias. However, a caveat is that the patients in the latter study had an inadequate response to D2 antagonists and so may have been refractory or at a ceiling of efficacy, accounting for the lack of augmentation with the addition of the PDE10A inhibitor. Thus, it will be of interest to further explore the therapeutic potential of more indirect pathway-biased PDE10A inhibitors, if such can be developed, or to investigate the combination of a PDE10A inhibitor with a low dose of D2 antagonist in acute exacerbation patients.

The discussion above is focused on comparison of D2 antagonists and PDE10A on molecular aspects of signaling. Orthogonal to this is a comparison based on behavioral effects in preclinical models believed to be predictive of antipsychotic efficacy. Particularly, PDE10A inhibitors are very effective at inhibiting NMDA receptor channel blocker-induced hyperlocomotor activity and at blocking conditioned avoidance responding in rodents, activities shared with D2 receptor antagonists. In humans, NMDA receptor channel blockers cause behavioral effects remarkably similar to those exhibited by humans with schizophrenia (Luby et al., 1959; Krystal et al., 2003; Javitt et al., 2012). Accordingly, the ability of PDE10A inhibitors to effectively block hyperactivity induced by NMDA receptor channel blockers in rodents was significantly supportive for advancing this class into clinical trials as a therapeutic for schizophrenia. However, the mechanisms by which channel blockers are “schizophrenomimetic” in humans (Luby et al., 1959) or induce hyperactivity in rodents are not well-understood, nor is the mechanism by which PDE10A inhibitors, or D2 antagonists, block their effects in rodents beyond the hypothesis that both activate indirect pathway. Thus, at present, there are limited back-translational learnings from the failure of the PDE10A inhibitors to evidence clinical antipsychotic activity, other than that blockade of channel blocker induced hyperactivity is apparently not predictive of therapeutic efficacy.

A more significant finding supporting the investigation of PDE10A inhibitors as antipsychotics was their very effective blockade of conditioned avoidance responding in rodents. Psychotic and delusional symptoms in schizophrenia are hypothesized to arise from aberrant dopamine signaling resulting in mis-attribution of stimulus salience (Kapur et al., 2005; Winton-Brown et al., 2014). Conditioned avoidance responding is a rodent behavioral assay of stimulus salience (Wadenberg, 2010). D2 antagonists are very effective at blocking conditioned avoidance responding and this effect is interpreted to reflect the ability of these compounds to dampen psychosis and delusions in patients with schizophrenia by dampening the aberrant attachment of salience to innocuous sensory cues. Contributing a strong element of predictive validity to the assay, compounds from a number of pharmacological classes that failed to inhibit conditioned avoidance responding in rodents also failed to prove antipsychotic in clinical trials. Thus, the ability of PDE10A inhibitors to reduce stimulus salience in the rodent assay was one of the strongest considerations driving the clinical development of these compounds as antipsychotics. Nonetheless, PDE10A inhibitors have not been found to be effective antipsychotics. We offer a possible framework for interpreting this lack of translation. Despite the behavioral phenocopy, it is possible that PDE10A inhibitors suppress conditioned avoidance responding by altering a basal ganglia computation that is distinct from that by which D2 receptor antagonists suppress this behavior. This interpretation is prompted by the findings from the primate studies of Papa and colleagues contrasting effects of the D2 receptor antagonist risperidone and the PDE10A inhibitor MP-10 on a Kluver Board reaching task (Uthayathas et al., 2014). Both compounds disrupted performance; however, risperidone appeared to disrupt motor functions necessary to perform the task without an apparent effect on motivation to perform, whereas MP-10 appeared to impact motivation or the reward value of task performance without impacting the motor ability to perform. Regardless of exact overt behavioral constructs, the effects of PDE10A inhibitors and D2 receptor antagonists on basal ganglia computations is evidently different based on the effects on primate behavior, yet this difference nonetheless yields a behavioral phenocopy in rodent measures such as conditioned avoidance responding. The important point is that whatever the effect of PDE10A inhibition on basal ganglia computation, it is not antipsychotic.

The preceding section of the Discussion outlined a number of key differences between PDE10A inhibitors and D2 antagonists. Unfortunately, it is not clear which, if any, of these are responsible for the difference in clinical antipsychotic efficacy. Nonetheless, we hope this part of the review provides some initial triangulation points for investigating the basis for the differential efficacy. Next we outline some gaps in our knowledge regarding the physiology of PDE10A that may further serve in this regard and as also as starting points for developing new therapeutic uses for PDE10A inhibitors.

The fact that PDE10A inhibition impacts both direct and indirect pathway function suggests that the consequences of PDE10A inhibition may more fruitfully be investigated with respect to their effects on integrated outputs of the direct and indirect pathways acting in concert rather than in opposition. Recent analyses of basal ganglia information processing highlight direct and indirect pathway co-activation and co-ordination during behavior integration (Calabresi et al., 2014; Cox and Witten, 2019). In particular, several groups have found the direct and indirect pathways are activated in concert, not in opposition, at the initiation of movement and action selection in mice (Cui et al., 2013; Tecuapetla et al., 2016; London et al., 2018). Given that PDE10A inhibitors activate the direct and indirect pathways in concert, analyses of their effects may be better framed by what is being learned about how the two MSN populations function as a single network to integrate information. In fact, PDE10A inhibitors may provide an important tool to study such integration. However, the behavioral effects of PDE10A inhibitors suppress action selection, not facilitate this activity as may have been predicted if PDE10A inhibitors promote the concurrent activation of the direct and indirect pathways. This puzzle provides a segue to gaps in our knowledge regarding the effects of PDE10A inhibitors on two key aspects of the basal ganglia computational machinery, timing and plasticity.

An essential aspect of information processing by MSNs is the temporal integration of the corticostriatal input with dopamine signaling. Dopamine signaling has both tonic and phasic aspects (Goto et al., 2007). The timing of phasic dopamine signaling is critical to the assignation of reward value to ensembles of cortical inputs to MSNs as well as to the re-activation of the rewarded ensembles for subsequent action selection (Arbuthnott and Wickens, 2007). Given that the canonical function of phosphodiesterases is to regulate the timing and spatial spread of cyclic nucleotide signaling, PDE10A inhibition undoubtably has an effect on the temporal integration of signaling in MSNs. In one study relevant to this point, Yagishita et al. (2014) reported a role for PDE10A in regulating the timing of PKA activation on a sub-second time scale in distal dendrites of MSNs. PDE10A inhibition disrupted this critical timing and thereby degraded the specificity of the information signaled by cortical input in this compartment. Thus, one avenue for translational research is a more in-depth comparison of the effects of PDE10A and D2 receptor inhibition on short-time scale integration of information by striatal MSNs and the consequences to behavior.

At the other extreme of timing, D2 antagonists are administered chronically, and efficacy as currently measured in clinical trials emerges only after weeks of treatment. Furthermore, long term treatment with these agents induce significant long-time scale changes in striatal information processing, with a clear example being the induction of tardive dyskinesias (Jeste and Caligiuri, 1993). PDE10A inhibitors have a profound effect on gene expression in the MSNs (Kleiman et al., 2011). Such effects may be presumed to impact the functionality of these neurons with chronic treatment over long timescales. However, has not been explored for PDE10A inhibitors or for the effects of such compounds in comparison with D2 antagonists. In so far as such long-term effects contribute to the clinical efficacy of D2 antagonists, such studies may yield valuable insight into mechanisms of antipsychotic action.

In the same vein, different forms of synaptic plasticity are also essential to information processing by MSNs (Calabresi et al., 2007; Surmeier et al., 2009; Wickens, 2009; Lovinger, 2010). Given that cyclic nucleotide signaling is a key regulator of this plasticity (Calabresi et al., 2000), it is undoubtable that PDE10A inhibition impacts these processes. However, this aspect of PDE10A physiology and pharmacology has not yet been studied in depth. Elucidating the effect of PDE10A inhibition on the multiple forms of corticostriatal synaptic plasticity would provide a valuable reference point in inferring how PDE10A inhibitors impact information processing by striatal MSNs. Again, comparison of PDE10A and D2 inhibition in this regard may serve as another point of triangulation for understanding the differences in antipsychotic efficacy.

The gaps in our knowledge regarding the effects of PDE10A inhibitors highlighted above focus on molecular mechanisms. However, perhaps the most significant gap in our knowledge regarding PDE10A inhibitors as well as D2 receptor antagonists is a clearer understanding of the effects of such compounds in humans, both in healthy individuals and those suffering from schizophrenia. There is currently no validated method to assess activation of the indirect striatal output pathway in humans. Nonetheless, advances in functional imaging and the determination of regional connectivity are beginning to shed light on the circuitry that may be dysfunctional in schizophrenia (Tarcijonas and Sarpal, 2019). Reduced corticostriatal connectivity has been associated with psychosis and clinical improvement with antipsychotic therapy is associated with improved connectivity between specific cortical regions and the striatum (Sarpal et al., 2015). Although this effect cannot be definitively localized to indirect pathway neurons, as these neurons express the majority of D2 receptors in the striatum, they are likely to be a significant contributor to the imaging signals. If an increase in cortico-striato-pallidal connectivity is a biomarker of the clinical efficacy of D2 antagonists, the lack of clinical efficacy with PF-2545920 and TAK-063 predicts such connectivity will not be improved by these compounds. Alternatively, enhanced cortico-striatal connectivity by these compounds similar to that caused by D2 receptor inhibition would indicate that improved connectivity alone is not sufficient for a therapeutic response or suggest that PDE10A inhibition uniquely produces additional circuitry effects that confound this benefit. Thus, although PDE10A inhibitors will not be a treatment for schizophrenia, they may still be useful clinical tools in understanding the disorder and in the development of new biomarkers of efficacy and medications.

A simple but essential complimentary step to imaging studies such as discussed above is an in-depth clinical evaluation of the *subjective* effects of PDE10A inhibition in humans. Despite the fact that multiple PDE10A inhibitors have been tested in humans, we lack fundamental information on their subjective effects due to the requirements for conducting and blinding Phase I and Phase II clinical studies. This leaves us to infer behavioral consequences, as on “stimulus salience” or “action selection,” from animal data. Obviously, our inferences that PDE10A inhibitors may be antipsychotic based on the animal data were wrong. Given that there are a number of PDE10A inhibitors that have proven to be safe and well-tolerated in humans, our strong recommendation is the conduct and publication of studies on the subjective effects of PDE10A inhibition in people. Ideally, this study would include a D2 receptor antagonist as comparator. A model for this analysis may be the study of Papa and colleagues in rhesus monkeys (Uthayathas et al., 2014). This would be a straightforward way to gain insight into the significance of the differential effects of TAK-063 and PF-02545920 on measures of global clinical impressions observed in the Phase II studies. Such a study may also provide valuable insight into the different cognitive domains tapped by these clinical global measures in comparison to the PANSS. This will provide an essential foundation for framing further back-translational behavioral studies and for interpreting the effects of these compounds on behavior at the molecular level, on the way to developing new and better treatments for schizophrenia and related disorders. Finally, such studies may serve as an important step in considering alternative clinical indication for PDE10A inhibitors and to capitalize on the tremendous investment that has been made in the novel pharmacology.

## Summary and Conclusion

Why are D2 antagonists antipsychotic? Nearly 70 years after the first clinical use of chlorpromazine we do not have enough of a molecular understanding to design mechanistically new drugs that have similar, let alone better, efficacy. A potentially powerful approach toward gaining such understanding is the back-translational comparison of the effects of D2 antagonists with different pharmacologies that have been tested in the clinic but failed to evidence comparable antipsychotic efficacy. In this regard, we suggest that PDE10A inhibitors may be particularly useful because of the enzyme's very restricted distribution to striatal MSNs and the relatively straightforward effect of inhibitors to increase cyclic nucleotide levels in these neurons. There is already a wealth of published data on the effects of PDE10A inhibitors, reviewed here, that may enable back-translational efforts. Nonetheless, there remain significant gaps, notably on the effects of PDE10A inhibitors in humans, both healthy and suffering psychosis. The pharmaceutical industry has invested tremendously in the development of high quality PDE10A inhibitors. Rather than consider these efforts a “failure,” we suggest using these tools to continue to gain insight into the molecular basis for antipsychotic efficacy. Such work will undoubtedly aid in the development of new, more efficacious, safer antipsychotic agents and, indeed, may even provide insight into the nature of psychosis.

## Author Contributions

All authors contributed to the preparation of this review.

## Conflict of Interest

TC is currently employed by Pfizer Inc., CS is recently retired from Pfizer Inc., and FM was previously employed by Pfizer Inc. All authors while at Pfizer Inc. participated in the discovery and development of PF-0245920, a PDE10A inhibitor mentioned in the manuscript. However, the review article was prepared independently and the authors will accrue no commercial or financial benefit from publication that may be construed as a conflict of interest.

## Footnotes

^1^When microwave irradiation is used to rapidly inactivate tissue enzymatic activity.

^2^In this case measured by microdialysis.

^3^A similar conclusion can be drawn regarding PDE10A and the cyclic nucleotide pools regulated by D1 receptors in direct pathway MSNs.
